# Ureteral inflammatory edema grading clinical application

**DOI:** 10.3389/fsurg.2022.1038776

**Published:** 2023-01-06

**Authors:** Jialin Li, Chengming Jiang, Xinzhi Liao, Sheng Yan, Sigen Huang, Shengyin Liu, Quanliang Liu

**Affiliations:** ^1^ The First Clinical College, Gannan Medical University, Ganzhou, China; ^2^ Department of Urology, The First Affiliated Hospital of Gannan Medical University, Ganzhou, China

**Keywords:** ureteral inflammatory edema, upper urinary calculi, endoscopic surgery, clinical management, classification

## Abstract

**Purpose:**

To evaluate the relationship between endoscopic ureteral inflammatory edema (UIE) and ureteral lumen, formulate a preliminary grading method for the severity of UIE, and analyze the impact of different grades of UIE on endoscopic ureteral calculi surgery and prognosis.

**Materials and methods:**

We retrospectively analyzed 185 patients who underwent ureteroscopic lithotripsy (URSL) for upper urinary tract stones between January 2021 and November 2021. The UIE grade and lumen conditions were assessed by endoscopic observation. The effect of UIE grade on URSL and on patient prognosis were analyzed by multiple linear regression and binary logistic regression.

**Results:**

A total of 185 patients were included in the study. UIE grade showed a significant correlation with age, hydronephrosis grading (HG), ureteroscope placement time (UPT), surgery time (ST), hemoglobin disparity value (HDV), and postoperative ureteral stenosis (PUS) (*P *< 0.05). Logistics regression analysis showed a gradual increase in intraoperative UPT and ST with increase in UIE grade. The severity of UIE showed a negative correlation with improvement of postoperative hydronephrosis (IPH) and the appearance of PUS. HDV was significantly increased in patients with UIE grade 3.

**Conclusions:**

UIE grading can be used as an adjunctive clinical guide for endoscopic treatment of upper urinary tract stones. The postoperative management measures proposed in this study can help inform treatment strategy for ureteral stones.

## Introduction

Ureteral inflammatory edema (UIE) is one of the main factors that hinder ureteroscopic lithotripsy (URSL), and leads to various complications such as prolonged surgery time (ST), reduced calculi-free rate, and postoperative ureteral stenosis (PUS) in patients with ureteral calculi (1–3). Over the last 30 years, there has been a gradual increase in reports of ureteral calculi complicated by UIE, which is mainly caused by irritation of the ureteral wall by the stone and other factors that induce an inflammatory response (4). Since the imaging features of UIE mimic those of pelvic-ureteral junction obstruction and simple upper ureteral calculi, preoperative diagnosis of upper ureteral calculi combined with UIE is relatively difficult. Therefore, intraoperative ureteroscopy is the best way for identification, histological diagnosis, and treatment of ureteral stones combined with inflammatory edema (5, 6).

UIE associated with calculi usually causes renal impairment with significant obstructive symptoms. Currently, the surgical treatment for UIE is mainly based on the degree of edema and the presence of exacerbating factors and is performed in combination with ureteroscopy. However, the presence of large-scale UIE and ureteropelvic junction stenosis in patients with ureteral stones may necessitate different surgical approaches, such as laparoscopic or robotic surgery, but there are no clear criteria for selecting the surgical approach (7). The classification of ureteral calculi combined with inflammatory edema has not been reported in the literature. Therefore, the aim of this study was to evaluate and grade UIE by endoscopic examination and to analyze the impact of different grades of UIE on the management of upper urinary tract calculi during URSL and patient prognosis.

## Materials and methods

### Study population

We enrolled patients who received URSL (including flexible ureteroscope group and rigid ureteroscope group) for upper urinary tract stones between January and December 2021 at the Department of Urology, First Affiliated Hospital of Gannan Medical University. The surgical procedures are described in supplementary materials. The study complied with the Declaration of Helsinki and was approved by the Institutional Ethics Committee of the First Affiliated Hospital of Gannan Medical University. Informed consent was obtained from all patients prior to their enrolment. The flow chart of the study is shown in Supplementary Figure S1. The inclusion criteria were: (a) Patients with upper urinary tract calculi who were scheduled to undergo URSL preoperatively; (b) availability of complete clinical information; (c) provision of written informed consent. The exclusion criteria were: (a) Patients undergoing planned staged procedures; (b) history of ureteroscopic procedure within the preceding 3 months; (c) patients undergoing preoperative ureteral stent placement or preoperative nephrostomy. Data pertaining to a total of 200 patients with upper urinary tract calculi treated *via* ureteroscopy were collected, of which 185 patients were included in the analysis.

### Data collection

All data were obtained from the hospital medical records. The data collected include basic preoperative patient information and disease status. Intraoperative parameters such as ST, intraoperative complications, and residual calculus (RC) were recorded along with UIE from real-time photographic records during flexible ureteroscopy as well as rigid ureteroscopy procedures, respectively. We also recorded prognostic indices such as duration of hospitalization and pathological results of inflammatory edema biopsy.

### Related indices and definitions

**Figure 1:**
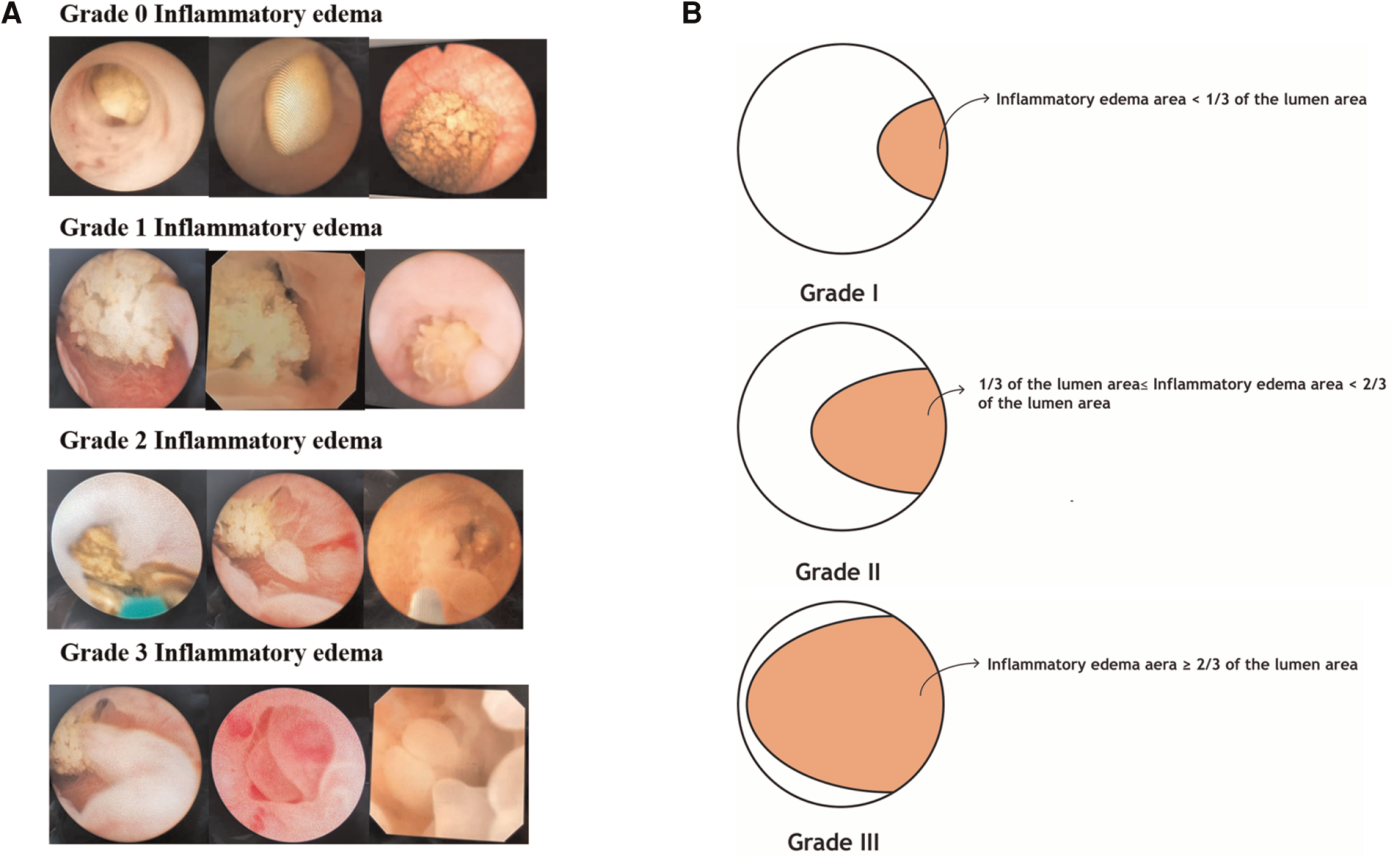
Ureteral inflammatory edema grading definition legend. (**A**) is a ureteroscopic view of inflammatory edema. (**B**) is a schematic diagram of ureteral edema grading.

In the present study, UIE was broadly graded by endoscopic observation into the following four grades (Figure 1): grade 0, no inflammatory edema; grade 1, inflammatory edema covering not more than 1/3 of the lumen in maximum cross-section; grade 2, inflammatory edema covering 1/3–2/3 of the lumen in maximum cross-section; and grade 3, inflammatory edema covering ≥2/3 of the lumen in maximum cross-section. For definition of clinical indices, see supplementary material.

### Statistical analysis

Data analyses were performed using SPSS (IBM 25.0) statistical software. Among the continuous variables, age, body mass index (BMI), calculi size, and surgery time showed normal distribution and were statistically analyzed using one-way analysis of variance (ANOVA). Ureteroscope placement time (UPT), hemoglobin disparity value (HDV), postoperative hospitalization time (PHT), and total hospitalization time (THT) showed a non-normal distribution and were analyzed using the non-parametric Kruskal–Wallis *H* test. Correlations between groups for sex, underlying medical history (UMH), ureteroscopy type (UT), location of calculi, hydronephrosis grading (HG), urine bacterial culture (UBC), PUS, RC, improvement of postoperative hydronephrosis (IPH), inflammation aggravated (IA), improvement in renal function (IRF), post-operative fever (POF), and UIE were statistically analyzed using the Chi-squared test. Multiple linear regression analysis and binary logistic regression were performed to analyze the effect of the severity of UIE and other factors on the prognosis of patients.

## Results

Out of 200 patients, 15 patients were lost-to-follow-up. Finally, 185 patients (97 male, 88 female; mean age: 47.9 ± 12.1 years; mean BMI: 23.9 ± 3.6 kg/m^2^) with complete follow-up were included in the analysis. The distribution of the inflammatory edema grade in the rigid ureteroscope group was as follows: grade 0, 24 cases; grade 1, 22 cases; grade 2, 17 cases; grade 3, 19 cases. The distribution of inflammatory edema grade in the flexible ureteroscope group was: grade 0, 33 cases; grade 1, 37 cases; grade 2, 19 cases; and grade 3, 14 cases. There was a significant association of UIE grade with age, HG, ST, UPT, HDV, and US (*P *< 0.05) (Table 1).

**Table 1:** General information of patients with ureteral inflammatory edema classification. Abbreviations: UMH, underlying medical history; HG: hydronephrosis grading; UBC, urine bacterial culture; UT, ureteroscopy type; ST, surgery time; UPT, ureteroscope placement time; HDV, hemoglobin disparity value; PUS, postoperative ureteral stenosis; RC, residual calculi; IPH, improvement of postoperative hydronephrosis; IRF, improvement of renal function; PHT, postoperative hospitalization time; THT, total hospitalization time; IA, inflammation aggravated; POF, postoperative fever.

	Ureteral inflammatory edema grading				*P* value
	Grade 0	Grade 1	Grade 2	Grade 3	
Age (year)	45.2 ± 12.3	47.5 ± 10.4	52.3 ± 13.9	47.8 ± 11.7	0.028*
UMH					0.556
Yes	16	15	6	10	
No	41	44	30	23	
Sex	0.078				
Male	36	25	16	20	
Female	21	34	20	13	
BMI (kg/m^2^)	23.6 ± 3.3	23.5 ± 3.7	24.3 ± 3.8	24.3 ± 4.2	0.621
Calculi size (mm)	12.2 ± 5.9	12.7 ± 4.9	12.3 ± 4.85	13 ± 4.7	0.901
Location of calculi					0.379
Intra-renal	14	20	7	4	
Upper ureter	27	19	18	14	
Middle ureter	11	14	6	10	
Lower ureter	5	6	5	5	
Side					0.068
Left	21	34	20	20	
Right	36	25	16	13	
HG					<0.001*
None	3	2	2	0	
Mild	47	36	20	11	
Moderate	4	16	8	8	
Severe	3	5	6	14	
UBC					0.204
Positive	42	46	24	29	
Negative	15	13	12	4	
UT					0.288
Flexible ureteroscope	24	22	17	19	
Rigid ureteroscope	33	37	19	14	
ST (min)	46.8 ± 24.3	61.7 ± 27.2	60.0 ± 29.3	72.5 ± 36.9	0.001*
UPT (min)	3 (1,6.1)	4 (2,10)	5 (2,10.2)	4 (2,9.3)	<0.001*
HDV (Bleeding volume)	6 (0,23.4)	3 (0,24)	6 (0,22.4)	0 (0,19)	0.026*
PUS					<0.001*
Yes	1	2	5	9	
No	56	57	31	24	
RC					0.497
Yes	9	10	6	2	
No	48	49	30	31	
IPH					0.275
Yes	22	20	15	18	
No	35	39	21	15	
IRF					0.944
Yes	2	1	15	1	
No	55	58	21	32	
PHT (day)	2 (1,4.3)	2 (1,5)	2.5 (1,10)	2 (1,6.5)	0.235
THT (day)	6 (2.9,14.1)	7 (3,11)	7.5 (2,16.2)	6 (2,23.8)	0.538
IA					0.335
Yes	51	56	35	29	
No	6	3	1	4	
POF					0.321
Yes	8	4	7	4	
No	49	55	29	29	

* Statistically significant *P* value (*P *< 0.05).

To control for potential confounding factors, multiple linear regression was used to further analyze the effects for continuous variables, and binary logistic regression analysis was used to analyze the dichotomous categorical variables (Tables 2, 3). The results showed a significant association of UIE grade was with ST, UPT, and HDV. Moreover, UIE grade [*P *< 0.05, odds ratio (OR) = 2.85] was a significant factor affecting the PUS and IPH.

**Table 2:** Multiple linear regression for controlling the confounding factors in the study, the variables were further analyzed by multiple linear regression. Abbreviations: ST, surgery time; UPT, ureteroscope placement time; HDV, hemoglobin disparity value; PHT, post-operative hospitalization time; THT, total hospitalization time.

Covariate	Standardization coefficient *β*	*P* value
Age (year)	2.22	0.306
BMI (kg/m^2^)	0.69	0.742
ST (min)	0.27	0.000*
UPT (min)	0.42	0.002*
HDV	0.19	0.021*
PHT (day)	−0.07	0.374
THT (day)	−0.03	0.699

* Statistically significant *P* value (*P *< 0.05).

**Table 3:** Binary logistic regression for controlling the confounding factors in the study, the variables were further analyzed by binary logistic regression. Abbreviations: PUS, postoperative ureteral stenosis; RC, residuals calculus; IPH, improvement of postoperative hydronephrosis; IA, inflammation aggravated; IPF, improvement of renal function; POF, postoperative fever.

Covariate	Odds ratio*	95%CI	*P* value
PUS	2.85	1.32–6.17	0.008*
RC	0.99	0.49–2.01	0.990
IPH	0.19	0.08–0.43	0.000*
IA	0.91	0.50–1.67	0.770
IRF	0.75	0.30–1.89	0.540
POF	1.25	0.79–1.99	0.346

* Statistically significant *P* value (*P* < 0.05).

**Figure 2:**
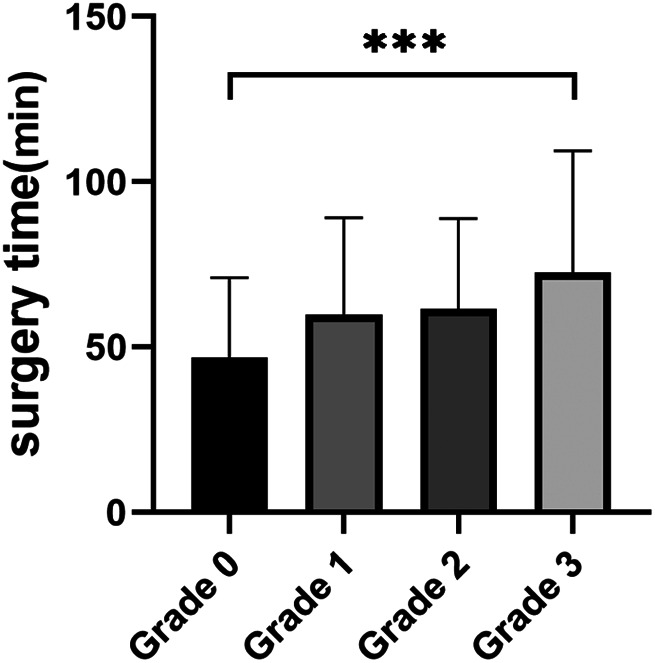
Statistical chart of postoperative stenosis rate.

**Figure 3:**
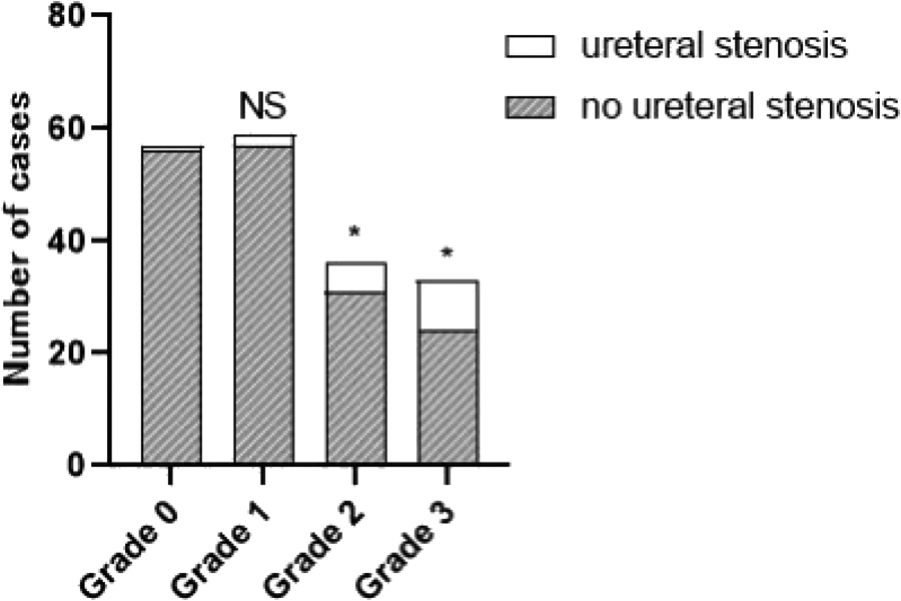
Statistical chart of surgery time.

Subsequently, we evaluated the correlation between UIE grade and a series of variables, based on the significant factors in the univariate analysis (Table 4). In this cohort, UIE was mostly found in the left ureter, and with the progressive increase of UIE grade, the surgery time was prolonged (Figure 2). The HDV increased significantly at UIE grade 3, and this indirectly increased the risk of surgery. In addition, UIE grade showed a negative correlation with IPH and the presence of PUS. Specifically, we observed a significant increase in the rate of PUS in patients with UIE grade ≥2 (Figure 3).

**Table 4:** Parameters associated with ureteral inflammatory edema. Abbreviations: UBC, urine bacterial culture; UMH, underlying medical history; UT, underlying medical history; PUS, postoperative ureteral stenosis; RC, residual calculi; IPH, improvement of postoperative hydronephrosis; IA, inflammation aggravated; IRF, improvement of renal function; POF, postoperative fever; ST, surgery time; UPT, ureteroscope placement time; HDV, ureteroscope placement time; PHT, post-operative hospitalization time; THT, total hospitalization time; HG, hydronephrosis grading.

	Edema (grade1)			Edema (grade2)			Edema (grade3)		
	OR	(95%CI)	*P* value	OR	(95%CI)	*P* value	OR	(95%CI)	*P* value
**(a) Dichotomous variables**									
Sex	2.33	(1.11–4.92)	0.026*	2.14	(0.92–5.01)	0.079	1.11	(0.46–2.69)	0.810
UBC	0.79	(0.34–1.86)	0.590	1.40	(0.56–3.48)	0.469	0.39	(0.12–1.28)	0.120
UMH	0.87	(0.38–1.99)	0.748	0.51	(0.18–1.46)	0.212	1.11	(0.44–2.85)	0.822
UT	0.82	(0.39–1.72)	0.596	1.23	(0.53–2.85)	0.628	1.87	(0.78–4.44)	0.159
Side	0.43	(0.20–0.90)	0.026*	0.47	(0.20–1.09)	0.079	0.38	(0.16–0.92)	0.031*
PUS	1.97	(0.17–22.28)	0.586	9.03	(1.01–80.8)	0.049*	21.00	(2.52–175.10)	0.005*
RC	1.11	(0.42–2.98)	0.834	1.07	(0.35–3.30)	0.911	0.34	(0.07–1.70)	0.191
IPH	0.82	(0.38–1.74)	0.599	1.14	(0.49–2.66)	0.768	1.91	(0.80–4.55)	0.144
IA	2.20	(0.52–9.24)	0.283	4.12	(0.48–35.71)	0.199	0.85	(0.22–3.27)	0.817
IRF	0.47	(0.04–5.38)	(0.547)	0.79	(0.07–8.99)	0.846	0.86	(0.07–9.86)	0.903
POF	0.45	(0.13–1.57)	0.209	1.48	(0.49–4.50)	0.491	0.85	(0.23–3.05)	0.797
**(b) Continuous Variables**									
Age (years)	2.28	(−2.10 to 6.66)	0.306	7.71	(2.69–12.73)	0.003*	2.64	(−2.52 to 7.79)	0.314
BMI (kg/mm^2^)	−0.24	(−1.59 to 1.11)	0.724	0.63	(−0.93 to 2.18)	0.426	0.57	(−1.03 to 2.16)	0.485
Calculi size (mm^2^)	0.50	(−1.41 to 2.41)	0.605	0.18	(−2.01 to 2.37)	0.872	0.79	(−1.46 to 3.04)	0.489
ST (min)	14.92	(4.39–25.46)	0.006*	13.17	(1.09–25.34)	0.033*	25.71	(13.30–38.11)	<0.001*
UPT (min)	1.59	(0.81–2.37)	<0.001*	1.68	(0.79–2.57)	<0.001*	1.96	(1.04–2.88)	<0.001*
HDV (Bleeding volume)	−0.37	(−3.11 to 2.37)	0.792	0.08	(−3.05 to 3.22)	0.958	−3.82	(−7.04 to 0.49)	0.021*
PHT (day)	−0.26	(−0.83 to 0.31)	0.374	0.69	(0.03–1.35)	0.039*	0.14	(−0.53 to 0.82)	0.675
Total hospitalization time (day)	−0.27	(−1.63 to 1.09)	0.699	0.87	(−0.69 to 2.43)	0.271	0.61	(−0.99 to 2.21)	0.456
**(c) Ordered multi-categorical variables**									
HG	0.07	(0.03–0.19)	<0.001*	0.22	(0.09–0.49)	<0.001*	0.26	(0.10–0.64)	0.004*

* Statistically significant *P* value (*P* < 0.05).

## Discussion

The incidence of ureteral calculi is still at a high level (8). Ureteroscopy is one of the main treatment options. Although ureteroscopy is a safe, minimally-invasive and efficient treatment modality, the postoperative prognosis is affected by several factors, such as calculi size, location, UIE, and the experience of the urologist (9, 10). In this study, we investigated the impact of different levels of UIE on calculi surgery by analyzing information about patients who underwent URSL.

The natural physiology of the ureter leads to a high incidence of calculi, and the associated complications of urinary obstruction and infection lead to impaired renal function (11, 12). During calculi surgery, a guidewire is used to pass through the area between the stone and the ureteral wall. However, in patients with inflammatory UIE, passage of the guidewire may lead to ureteral perforation or tear, which ultimately affects the removal of the stone and increases the risk of surgery (13).

Therefore, we considered UIE as an important factor affecting ureteroscopic calculi surgery. However, there is no relevant literature for evaluation and grading of UIE. During surgery, UIE is mostly located underneath the urinary stone, resulting in a restricted field of vision. Professor Gu et al. showed that UIE increases the risk of ureteral injury during holmium laser lithotripsy *via* ureteroscopy (9). With the widespread use of ureteral dilators, the ability to dilate the ureter and its lumen by injecting saline into the lumen of the ureter allows for clearer visualization of the narrowing at the site of the stone. At this point, for upper urinary tract stones, one of the reasons affecting surgical failure may be the size of UIE. The greater the inflammatory edema, the greater is the probability of surgical failure (13).

In this study, we noted significant association (*P *< 0.05) of UIE grade with age, ST, UPT, HDV, HG, and PUS. In addition, we also noted a corresponding increase in the time required for double-J tube placement with increasing grade of inflammatory edema (data not presented), suggesting that due care should be taken to prevent postoperative stenosis at the site of edema in patients with high-grade ureteral edema. We further noted that in most cases, the UIE was found on the left side of the ureter, which is consistent with the fact that stones are mostly found on the left side of the ureter, indicating a mutually reinforcing relationship between stones and inflammatory edema (14). This phenomenon was observed in 75.7% patients with UIE in this study, which further supports the existence of a close association.

Hydronephrosis as a preoperative factor of ureteroscopy has been observed in patients with stones with concomitant UIE (15). Most of these cases are prone to preoperative impaction, which in turn further worsens hydronephrosis (16). The potential underlying mechanism involves chronic stimulation of the ureteral wall by upper urinary tract stones leading to proliferation and infiltration of inflammatory cells. This causes thickening of the wall, subsequently aggravating the stenosis leading eventually to complications such as hydronephrosis (17). Moreover, according to our data, high-grade UIE is more likely to cause PUS, which also limits the recovery of renal function in patients with hydronephrosis.

In URSL, ST and UPT often affect the overall outcome of the stone, and more rapid completion of the operation can lessen the surgical trauma and improve prognosis (18, 19). In our study, UIE exceeding 1/3 of the ureteral lumen (i.e., UIE grade 2) was found to significantly affect the ST and UPT, and this effect was further increased with increase in the severity of edema, such as the emergence of mucosa-stone adherence (MSA). When the UIE is severely adherent to the stone, it can cause ureteral wall edema and inflammation, which may aggravate with ureteral peristalsis, resulting in a severely limited microscopic view and difficulty in separating the stone mucosa, indirectly increasing the complexity of the surgery and the risk of anesthesia and infection (15, 20). In addition, we also noted a significant increase in interoperative bleeding in patients with UIE grade 3. However, none of the cases in our study required blood transfusion. This suggests due attention should be paid to prevent HDV during surgery in such patients.

Our findings suggest that the higher the grade of UIE, the greater is the surgical risk (in terms of increased HDV and surgical difficulty) and the worse is the prognosis, while URSL in the setting of low-grade inflammatory edema is more beneficial with respect to improvement of renal function. In addition, patients with stones in the presence of UIEs, especially those with higher grade inflammatory edema, should be followed up for the occurrence of upper urinary tract calculi and treated with appropriate interventions at an early stage of stone formation. Whereas in patients with already combined calculi, URSL should be performed at an early stage to avoid surgical failure with increasing calculi. Further, for patients with grade 0 UIE, regular postoperative follow-up of renal function along with imaging should be performed to check for recurrence of calculi, signs of new inflammation and inflammatory irritants, or the presence of stenosis. For grade 1 UIE, along with postoperative follow-up of renal function and residual calculi, the focus should be on checking any increase in the degree of inflammatory edema or ureteral stenosis. Patients with ≥grade 2 UIE have a higher risk of postoperative ureteral stenosis due to the presence of inflammatory edema. Therefore, besides checking the renal function and residual stones, the stenosis site should also be evaluated during the follow-up, and in case of any indication for surgery, further treatment should be performed (21). Also, the presence of microscopic edema, surface roughness, bleeding, infiltration, and other malignant manifestations should be checked, and biopsy should be performed to confirm the diagnosis, if necessary. Based on this preliminary study, UIE grading can provide insights for intraoperative treatment and postoperative management of patients with stones to some extent.

Some limitations of this study should be considered. This was a single-center study with a small sample size and short follow-up period. Moreover, we did not assess the potential presence of neoplastic lesions in inflammatory edema or performed routine postoperative urine exfoliation cytology. Larger multicenter studies with longer follow-up are required to obtain more definitive evidence. The stone size measured in the study was the maximum stone diameter, and no comprehensive analysis was performed for multiple stones. Lastly, the association between edema grade and the development of neoplastic lesions in inflammatory edema is an issue worth investigating.

## Conclusion

UIE grading can be used as a clinical guide for endoscopic treatment of upper urinary tract stones, such as for assessing the timing of surgery, ureteroscope placement time, postoperative hydronephrosis improvement, and predicting the risk of postoperative ureteral stricture.

## Data Availability

The original contributions presented in the study are included in the article/**Supplementary Material**, further inquiries can be directed to the corresponding author/s.
