# Supplementary material for: Ureteral inflammatory edema grading clinical application
Source: Front Surg. 2023 Jan 6;9:1038776. doi: 10.3389/fsurg.2022.1038776 (PMC9852048; doi:10.3389/fsurg.2022.1038776)
Supplement: Supplementary file 1 [file Datasheet1.docx]

Supplementary Material

# Supplementary Data

## Abbreviations Used

URI=Ureteral inflammatory edema; URSL=ureteroscopic lithotripsy; BMI=body mass index; CT=computed tomography; CTU=computed tomography urography; UMH=underlying medical history; HG: hydronephrosis grading; UBC=urine bacterial culture; UT=ureteroscopy type; ST=surgery time; UPT=ureteroscope placement time; HDV=hemoglobin disparity value; PUS=postoperative ureteral stenosis; RC=residual calculi; IPH=improvement of postoperative hydronephrosis; IRF=improvement of renal function; PHT=postoperative hospitalization time; THT=total hospitalization time; IA=inflammation aggravated; POF=postoperative fever.

## Surgical Procedures

The patient is anesthetized, positioned in a lithotomy position, and routinely disinfected. A WOLF flexible ureteroscope or a WOLF rigid ureteroscope is placed through the ureteral orifice, connected to a cold light source and a flushing device, and the monitor slowly enters the bladder under direct vision to observe the bladder ridge, followed by the bilateral ureteral openings, and finally the affected ureter. The ureteral orifice is entered via the ureteroscope operation hole under the guidance of the zebra guidewire, which is a clear sign of entry of the ureteroscope into the ureter. This helps avoid damage to the ureter and its surrounding tissues caused by blind entry. At this time, photographs showing the calculi and surrounding tissues are obtained with a cell phone or digital camera, and the time of microscopy is recorded. The holmium laser fiber is prepared, wiped with iodophor, and introduced through the operation channel and holmium laser lithotripsy is performed. Initially, a small energy is chosen for lithotripsy, with a frequency of 8-15 Hz and an energy of 0.5-1.5 J. The maximum energy does not exceed 20 W. The energy level can be adjusted according to the size and hardness of the calculi, and the fiber should be in close contact with the calculi during lithotripsy to reduce the energy consumption. When confronted with slightly larger fragments that are harder and not easily broken, these can be taken out of the body using calculi extraction forceps. Before the end of the procedure, the entire ureter is inspected to assess any bleeding, calculi remnants, calculi displacement, etc. After completion of lithotripsy, the operation time is recorded, and a zebra guidewire is left in place while exiting the ureteroscope. One double J-tube is placed retrogradely under the traction of the guidewire. Usually, the upper end of the double J-tube reaches the renal pelvis and the lower end is located in the bladder.

## Related indices and definitions

Ureteral stenosis: A GE Brightspeed 16-slice spiral CT machine and a Siemens Definition AS 64-slice 128-slice spiral CT machine were used to determine the ureteral stenosis by conventional 5-mm flat scan, 1.25-mm or 1-mm reconstruction, parallel multiplanar reconstruction, curved reconstruction, partial CT enhancement scan, and CTU imaging.

Calculi size: Maximum diameter of the calculi in the ultrasound or CT report.

Location of calculi: The method refers to the segmentation of ultrasonography (upper segment: the ureteral junction of the renal mons to the upper edge of the sacroiliac joint; middle segment: between the upper and lower edges of the sacroiliac joint; lower segment: the ureteral bladder opening at the lower edge of the sacroiliac joint).

Hydronephrosis grading: Mild hydronephrosis: separation of the collecting system is 14-20 mm, and the renal parenchyma and renal shape are normal; moderate hydronephrosis: the separation of the collecting system is 21-35 mm, and there is mild thinning of the renal parenchyma; severe hydronephrosis: the separation of the collecting system is >36 mm, and the renal cortex appears significantly thinned.

Intraoperative and postoperative bleeding: The amount of bleeding was estimated based on intraoperative and postoperative hemoglobin changes, with a 10 g drop in hemoglobin representing 400 mL of blood loss.

# Supplementary Figures and Tables

## Supplementary Figures


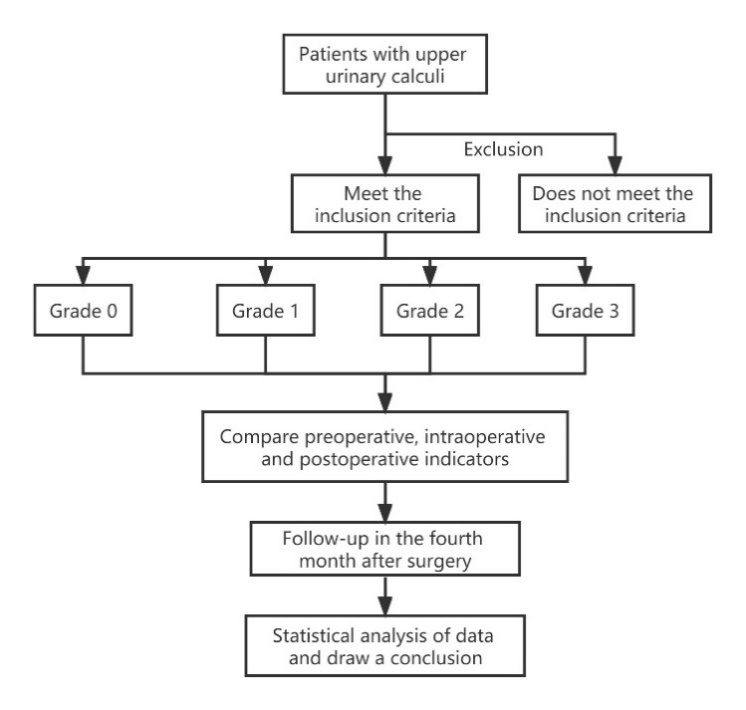


**Supplementary Figure 1.** **Technology Roadmap for Demonstration Studies**
